# How COVID-19 Outbreak Influenced Transplantation in Poland

**DOI:** 10.3390/jcm12020461

**Published:** 2023-01-06

**Authors:** Jacek Zawierucha, Tomasz Prystacki, Wojciech Marcinkowski, Jacek Stanisław Małyszko, Sławomir Nazarewski, Jolanta Małyszko

**Affiliations:** 1 Fresenius Medical Care Poland, 60-118 Poznan, Poland; 2 Fresenius Nephrocare Polska Sp. z o.o., 60-118 Poznan, Poland; 3 1st Department of Nephrology and Transplantology, Medical University of Bialystok, 15-540 Bialystok, Poland; 4 Department of General, Vascular and Transplant Surgery, Medical University of Warsaw, Banacha 1A, 02-097 Warsaw, Poland; 5 Department of Nephrology, Dialysis and Internal Medicine, Medical University of Warsaw, Banacha 1A, 02-097 Warsaw, Poland

**Keywords:** solid organ transplantation, stem cell transplantation, waiting list, qualification for transplantation, COVID-19 pandemic

## Abstract

Announced by the World Health Organization in early 2020, the pandemic caused by SARS-CoV-2 infections has had a huge impact on healthcare systems around the world. Local and international authorities focused on implementing procedures to safeguard the health of the population. All regular daily activities were disrupted. Similar factors related to the global fight against the COVID-19 epidemic also had a large impact on transplantation activity. In this article, the authors present the number of patients qualified for transplantation, transplanted and waiting on the waiting list in Poland during the 2-year period of the pandemic. In the first year of the epidemic (2020), all transplantation figures dropped drastically, by as much as 20–30% compared with 2019. The most disturbing fact is that the number of transplants performed in 2022 is still lower than before the outbreak of the epidemic (2019 and earlier).

## 1. Introduction

The COVID-19 pandemic had a large influence on medical services worldwide. Sanitary restrictions and additional hygiene procedures increased workloads and infections among professional personnel worsened the widespread shortage of medical staff in Polish healthcare providers and made access to physicians extremely difficult or, in most cases, limited the medical advice to contact over digital communication solutions (phone, computer, etc.). One of the most affected medical procedures was qualification for solid organ transplantation. Proper and successful qualification of potential organ recipients for transplantation requires completion of certain administrative and medical procedures such as selected lab tests (blood group, virology status, blood morphology, AST, ALT, GGLP, PTH, cholesterol concentration, HLA and immunogenetic tests, etc.), diagnostic imaging (i.e., lung X-ray, CT scan, coronarography in selected patients) and numerous consultations with specialists—transplant surgeon, gastroenterologist, cardiologist, dentist, etc. The detailed list of necessary checks is available at poltransplant.org. Even in the pre-pandemic period, completion of the required tests and consultations was one of the most time-consuming medical procedures in the country. For patients living outside of the big cities without access to specialized clinical centers, qualification for transplantation requires extreme effort in terms of travels, appointments, referrals etc. 

The aim of the research was to evaluate how the numbers of patients qualified for solid organ transplantation has changed in 2020 and 2021—the years of COVID-19 pandemic—compared with 2019 data, the last year without pandemic limitations and restrictions. Additionally, we compared the number of transplant procedures performed in the aforementioned period.

## 2. Landscape before the Outbreak

**Figure 1:**
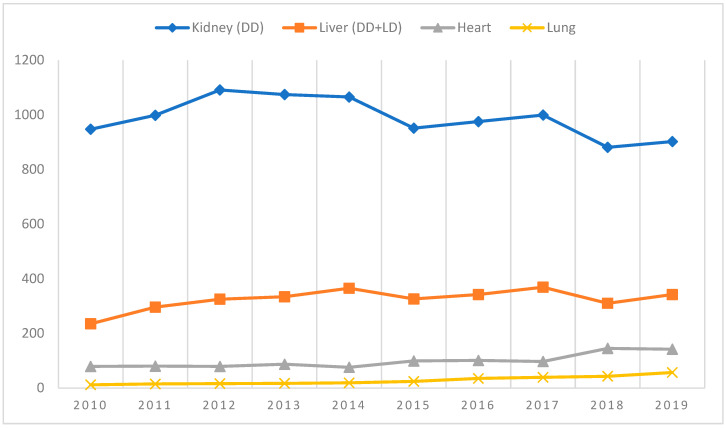
Number of solid organ transplantations in 2010–2019 [3].

Development of solid organ transplantation, as well as hematopoietic stem cell transplantation, has been observed in Poland for several decades [1,2,3,4]. The growing number of interventions and clinical centers has allowed us to look positively into the future. As shown in Figure 1, the number of SOTs (both deceased donors (DD) and living donors (LD)) was relatively stable with slight growth in the case of selected organs in the period 2010–2019. The outbreak of the COVID-19 epidemic completely changed the landscape, as the number of medical interventions fell and restoring the situation to how it was before will require a monumental effort on the part of medical providers.

## 3. Method

The official data from the Polish Transplant Coordinating Centre Poltransplant bulletins from 2019, 2020 and 2021 were analyzed in terms of the number of patients registered on the waiting list for solid organ transplantation [1,2,3,4].

The number of transplantations reported by Polish transplant centers was taken into consideration as well [1,2,3,4].

Additionally, we considered the number of stem cell transplantations in the discussed period [1,2,3,4].

## 4. Results 

### 4.1. Kidney (Deceased Donors)

Of all solid organ transplantations, kidney transplantation is the most common intervention worldwide. In Poland, the number of kidney Tx significantly exceeds all other forms of transplantation. As the most cost-effective in comparison with other types of renal replacement therapy, kidney transplantation remains the most desirable way of treating CKD. The COVID-19 pandemic had a large influence on all kinds of renal replacement therapy due to the numerous limitations put in place (shortage of medical staff, additional sanitary procedures, need to isolate infected patients).

As shown in Table 1, during the SARS-CoV-2 pandemic the number of qualified and transplanted patients decreased significantly. The worst affected data relates to new qualifications, which decreased about 30% in the first year of pandemic, as the effects of all the restrictions and limitations were felt. The second year of the pandemic (2021) brought better results in patients newly registered on the waiting list (+11.6%); however, it still remains at a level lower than in previous years (1178 in 2018 and 1160 in 2017).

The number of kidney Tx procedures performed during the 2-year pandemic period also decreased significantly and in the second year of the pandemic the negative trend continued (−0.8%). Indicative data from 2022 available on the Poltransplant webpage are also not optimistic: from January to September 2022, 553 kidneys were transplanted [4]. By extrapolation, we can assume that by the end of year the number of kidney transplantations will not exceed 800 procedures. These results differ significantly from previous years. In the years 2012–2016, around 1000 kidneys were transplanted annually in Poland, with 1091 kidney transplants performed in the best year 2012.

### 4.2. Pancreas, Kidney and Pancreas

Pancreas transplantation remains a debatable method of treating type I diabetes and in Poland it is limited to few interventions performed annually. Over the years (from 1988, when the first simultaneous kidney and pancreas transplantation was performed in Poland), the number of such interventions was around 20–30 procedures annually (the highest number was observed in 2012, when 43 pancreas alone or simultaneous kidney and pancreas transplantations were performed).

In 2020, pancreas transplantation (as well as isolated islets of Langerhans and kidney and pancreas transplantation) decreased dramatically. The same applies to new patients qualified for these interventions. Consequently, the number of active patients on the waiting list increased by about 40%. In 2021, the situation looked slightly better, with rapid growth of transplantations and stabilized numbers of active patients on the waiting list (Table 2).

### 4.3. Liver (Deceased Donors)

The second most common procedure in terms of numbers performed is liver transplantation. From 1989, when the first liver transplantation was successfully performed in Poland, the number of procedures gradually increased to several hundred in the twenty-first century. The SARS-CoV-2 pandemic halted this progress; compared to 2019—the last year free of COVID-19—figures in 2020 fell by more than 10% (Table 3). The number of liver transplantations was 15.5% lower than in the previous year. In turn, 2021 brought a little improvement in the number of procedures performed; however, they still remained at a lower level than before the pandemic. According to unofficial data published on the Poltransplant website in three quarters of 2022, 219 liver transplantations were performed. Extrapolation of this result would suggest that by the end of year the number will exceed 300 procedures, but that will still be lower than in the pre-pandemic period [4]. The total number of patients qualified for the treatment fell in the first year of the outbreak and increased slightly in the second. 

### 4.4. Lung

Lung transplantation remains the least performed solid organ transplantation (SOT) in Poland. The first operation was performed in 1997 and in the first subsequent decade there were no more than 10 patients transplanted in a single year. Significant growth in lung Tx has been observed in the last five years, where numbers increased by about 200% year on year. The period of the COVID-19 outbreak temporarily halted this development (−10.5% in the first year of pandemic); however, in 2021 the number of transplantations increased to 67 interventions with the positive trend continuing in 2022 with 61 lung transplantations performed from January to September (Table 4). As in previous years, the main indication for lung transplantation was complications in the course of cystic fibrosis, but in the last two years, complications after SARS-CoV-2 infection became a significant indication for such an intervention. 

### 4.5. Heart

From the first heart transplant performed in Poland in 1985 to the end of 2021, 3101 organs have been transplanted, which puts heart transplants in third place in terms of the number of procedures performed. In the last decade, the number increased from less than 100 to 200 in 2021. The COVID-19 outbreak had no influence on treatment numbers—in both years of the pandemic we observed growth in the number of qualified patients, as well as the number of transplants performed (Table 5). Heart transplantation is the only form of SOT which seems to be unaffected by the influence of the outbreak. As a life-saving procedure, which cannot be replaced by any other treatment, the growing trend continues. Even though the COVID-19 pandemic resulted in limited availability for the treatment, we observed constant growth in the case of heart transplantation. 

### 4.6. Living Donor Transplantation

Transplantation from living donors represents a small proportion of the total number of procedures performed. Although the first living donor kidney transplantation in Poland was performed in 1966, in subsequent decades the number of procedures never exceeded 10 Tx/year. It was not until 1996 that 12 kidneys were transplanted from living donors, and further development of this method could be observed in the 21st century, where the number of transplants reached 30–40 in one year. 

In the first year of the outbreak, living kidney donor transplantation fell dramatically (about 40% fewer interventions performed) and increased significantly in 2021 (+41% compared with 2020) (Table 6). We also see a positive trend in 2022. In the first nine months of 2022, transplant centers performed 53 living kidney donor transplantations, and the final number may reach 70 procedures [4]. It would be the best result in Polish history; however, it still remains below the population requirement, and the expectations and potential of transplant centers.

In 1999, the first living donor liver transplantation was performed. In subsequent years, the number of treatments gradually increased, although it never exceeded 30 procedures in a single year. The COVID-19 pandemic had no influence on the annual number of interventions. Figures from 2020 indicate growth for procedures performed, and in 2021 the number fell to the level seen in 2019. 

As the number of living donor transplantations remains at a very low level, the influence of the COVID-19 outbreak on the results is also limited, and it failed to change the overall landscape in a significant way.

### 4.7. Waiting Times for Solid Organ Transplantation

All restrictions and limitations connected with the COVID-19 outbreak greatly influenced waiting times for an organ. As shown in Table 7, reported waiting times changed (increased) significantly during the pandemic period compared with the year before the outbreak.

Waiting times are derived from the result of the number of patients qualified for transplantation, the number of organs taken for transplantation, and the number of procedures performed during the year. All three parameters were affected in the pandemic period. As shown above, the number of patients qualified and the number of interventions decreased significantly in 2020. Besides the number of patients qualified, the availability of potential donors seems to be a crucial factor influencing prolongation of waiting time. The most significant prolongation was observed in kidney transplantation (from 273 days in 2019 to 430 days in 2020) and heart transplantation (362 days vs. 603 days). 

A comparatively better situation was observed in the case of lung and liver transplantation. In the case of liver transplantation, waiting times were kept on a similar level during the 3-year observation period. Waiting times for lung transplantation were 62 days longer in 2020 and 48 days longer in 2021 than in 2020.

Major problems with donor availability and organ collection (organization of organ collection, discouraging formalities and bureaucracy) remained even after the pandemic period and decreasing waiting times has become the most serious challenge for solid organ transplantation development.

### 4.8. Stem Cell Transplantation

As in the case of solid organ transplantation, the COVID-19 outbreak halted stem cell transplantation (SCT) development in Poland. In 2020 the number of matched unrelated donors (MUD) SCTs—the most common intervention in Poland—decreased significantly (Table 8). Such a high drop may be linked to the risk of consequences of immunosuppression and the fatal course of SARS-CoV-2 infection in transplanted patients. Additionally, pandemic restrictions limited patients’ contact with their physicians and the availability of diagnostic tests required before transplantations. On the other hand, the pandemic period also influenced the availability of potential donors and the possibility of importing stem cell (bone marrow, peripheral blood) from different countries into Poland. 

It is remarkable that in 2021 the number of MUD SCTs grew rapidly and exceeded figures from previous years. 

## 5. Discussion

Solid organ transplantation is a well-developed medical intervention for millions of patients worldwide. For some patients, it remains the only chance of life, and for other it helps them to lead a normal life without the need for supporting machines (e.g., hemodialysis). It is well known that kidney transplantation—the most common kind of transplantation—significantly improves survival rates, reduces expenses, and enhances quality of life for patients with chronic kidney disease. Heart, lung, or liver transplantation in most cases represents a life-saving procedure for the majority of patients suffering with chronic heart failure, cystic fibrosis, or hepatic cirrhosis, for instance.

On the other hand, many barriers remain which limit the number of SOTs performed in the world. Besides the economic factors that depend on the country’s financial situation and willingness to pay to improve medical services, cultural, organizational, and religious rules play a significant role in the development of transplantation. The patient’s age, mobility, financial status, and awareness of different treatment options are also important. Psychological factors such as fear, anxiety, or guilt may also play an important role in the decision-making process. These personal factors greatly influence prompt referral and qualification for transplantation [5,6]. 

Proper qualification of patients for solid organ transplantation is an interdisciplinary process, which requires significant effort and time on the part of healthcare providers, as well as massive engagement from the patient. In most developed countries, the procedures precisely describe the qualification process, as well as the roles and responsibilities of every stakeholder. Usually, the procedures were developed on a national level, are maintained by national agencies responsible for transplantation, and may differ from country to country. In general, qualification for SOT should provide, where possible, a complete picture of the recipient’s actual health status and lead to a successful intervention. The major obstacle to complete qualification is mixed pathways (discrepancies caused by healthcare providers and sometimes an unclear flow of funds between selected providers). As the patient being prepared for transplantation belongs to part of the whole system, prioritization of healthcare services plays a key role in successful qualification. In situations of limited availability of selected procedures, the patient undergoing qualification sometimes has to wait in line or make use of private services to complete the required checks. 

The COVID-19 outbreak brought additional challenges for both healthcare providers and patients. Beside the sanitary regulations and physicians’ focus on continuity of service and urgent procedures, the lockdowns had an influence on patient behavior. A lot of papers published indicated the fear of being infected as a factor for avoiding any non-urgent contact with healthcare services [7,8,9,10]. On the other hand, in many countries the outbreak deepened the exclusion of communication, with mass communication (on public buses or trains) being subject to sanitary restrictions (number of occupied seats in public transport, reduction in the number of or cancellation of treatment courses) and making normal contact with physicians “mission impossible” in some cases. 

The unknown virus, unknown infection-related mortality and unknown methods of treatment at the beginning of the epidemic also influenced physicians’ behavior. Immunosuppression used during transplant procedures increases the risk of infection and can impair the development of antibodies in immunocompromised patients. After some investigations, it was found that transplantation patients using immunosuppressive agents can produce antibodies against SARS-CoV-2 [11], and this may have given physicians slightly more confidence in their therapeutic decisions. 

The improvement in the quality of care and restoration to almost normal activity has been observed when medical service providers developed procedures for service organization and risk management during the qualification and selection of donors and patients to be transplanted [12]. The real breakthrough came with the introduction of vaccination against SARS-CoV-2 and national vaccination programs. At-risk patient groups (patients on dialysis, patients qualified for transplantation, immunocompromised patients) were vaccinated in the first wave at the very beginning of the program. Successful vaccination programs decreased mortality, improved outcomes, and ultimately stabilized patients’ situations in the country [13]. The current situation in Poland, according to John Hopkins University data, is quite stable with 13,079 new cases reported in the last 28 days [14].

As shown in previous sections of this paper, all the factors listed above influenced the number of patients qualifying for solid organ transplantation (active on waiting lists) as well as the number of operations performed.

## 6. Conclusions

The period of the pandemic had a great impact on the development of transplantation in Poland. Restrictions caused by the pandemic have deprived many patients of any chance of treatment. We shall focus on the rapid growth in the number of transplants in the years following the pandemic. Nevertheless, the community of transplant specialists should introduce renewed cooperation with national authorities to implement the best possible procedures to protect patients referred for transplantation in the event of future epidemic situations.

## Figures and Tables

**Table 1:** Number of patients registered for kidney transplantation on the Poltransplant’s waiting list in 2019, 2020 and 2021 [1,2,3].

Year	2019	2020	2021	Delta 2020/2019 [*n*/%]	Delta 2021/2020 [*n*/%]
Total number of patients [*n*]	2747	2101	2058	−646/−23.5	−43/−2.0
New patients qualified [*n*]	1066	750	837	−316/−29.6	+87/+11.6
Active on waiting list (31 December) [*n*]	1165	1007	984	−158/−13.6	−23/−2.3
Transplanted [*n*]	902	714	709	−188/−20.8	−5/−0.8

**Table 2:** Number of patients registered for pancreas and kidney, islets of Langerhans and pancreas alone transplantation on the Poltransplant’s waiting list in 2019, 2020 and 2021 [1,2,3].

Year	2019	2020	2021	Delta 2020/2019 [*n*/%]	Delta 2021/2020 [*n*/%]
Total number of patients [*n*]	129	73	94	−56/−43.4	+21/+28.8
New patients qualified [*n*]	44	27	38	−17/−38.6	+11/+40.7
Active on waiting list (31 December) [*n*]	45	63	61	+18/+40.0	−2/−3.2
Transplanted [*n*]	34	4	20 *	−30/−88.2	+16/+400

* incl. one liver and pancreas transplantation.

**Table 3:** Number of patients registered for liver transplantation on the Poltransplant’s waiting list in 2019, 2020 and 2021 [1,2,3].

Year	2019	2020	2021	Delta 2020/2019 [*n*/%]	Delta 2021/2020 [*n*/%]
Total number of patients [*n*]	675	500	514	−175/−25.9	+14/+2.8
New patients qualified [*n*]	407	359	368	−48/−11.8	+9/+2.5
Active on waiting list (31 December) [*n*]	128	138	146	+10/+7.8	+8/+5.8
Transplanted [*n*]	341	288	293	−53/−15.5	+5/+1.7

**Table 4:** Number of patients registered for lung transplantation on the Poltransplant’s waiting list in 2019, 2020 and 2021 [1,2,3].

Year	2019	2020	2021	Delta 2020/2019 [*n*/%]	Delta 2021/2020 [*n*/%]
Total number of patients [*n*]	251	253	289	+2/+0.8	+36/+14.2
New patients qualified [*n*]	140	110	128	−30/−21.4	+18/+16.4
Active on waiting list (31 December) [*n*]	109	168	163	+59/+54.1	−5/−3.0
Transplanted [*n*]	57	51	67	−6/−10.5	+16/+31.4

**Table 5:** Number of patients registered for heart transplantation on the Poltransplant’s waiting list in 2019, 2020 and 2021 [1,2,3].

Year	2019	2020	2021	Delta 2020/2019 [*n*/%]	Delta 2021/2020 [*n*/%]
Total number of patients [*n*]	971	724	781	−247/−25,4	+57/+7.9
New patients qualified [*n*]	305	239	351	−66/−21.6	+112/+46.9
Active on waiting list (Dec 31) [*n*]	462	415	410	−47/−10.2	−5/−1.2
Transplanted [*n*]	141	145	200	+4/+2.8	+55/+37.9

**Table 6:** Living donor transplantation in 2019–2021 [1,2,3].

Year	2019	2020	2021	Delta 2020/2019 [*n*/%]	Delta 2021/2020 [*n*/%]
Kidney [*n*]	52	31	44	−21/−40.4	+13/+41
Liver [*n*]	21	28	20	+7/+33.3	−8/−28.6

**Table 7:** Waiting times for solid organ transplantation [1,2,3].

Year	2019	2020	2021	Delta 2020/2019 [days]	Delta 2021/2020 [days]
Kidney (first transplantation)					
urgent [days]	49	30	144	−19	+114
planned [days]	273	430	442	+157	+12
highly sensitized [days]	762	1222	899	+460	−323
Kidney (next transplantation)					
planned [days]	621	559	671	−62	+112
Pancreas [days]	363	509	438	+146	−71
Liver					
urgent [days]	7	1	29	−6	+28
planned [days]	128	121	134	−7	+13
Lung					
urgent [days]	19	1	217	−18	+216
planned [days]	163	225	273	+62	+48
Heart					
urgent [days]	59	91	298	+32	+207
planned [days]	362	603	405	+241	−198

**Table 8:** Number of allogeneic stem cell transplantations in 2019–2021 [1,2,3].

Year	2019	2020	2021	Delta 2020/2019 [*n*/%]	Delta 2021/2020 [*n*/%]
Related donors (MRD)	185	168	177	−17/−10	+9/+6
Unrelated donors (MUD)	423	386	484	−37/−9	+98/+25
Haploidentical	78	80	86	+2/+2.5	+6/+7.5

## Data Availability

The data presented in this study are available on request from the corresponding author.
